# Prophage excision switches the primary ribosome rescue pathway and rescue‐associated gene regulations in *Escherichia coli*

**DOI:** 10.1111/mmi.15003

**Published:** 2022-12-05

**Authors:** Haruka Onodera, Tatsuya Niwa, Hideki Taguchi, Yuhei Chadani

**Affiliations:** ^1^ School of Life Science and Technology Tokyo Institute of Technology Yokohama Japan; ^2^ Cell Biology Center, Institute of Innovative Research Tokyo Institute of Technology Yokohama Japan

**Keywords:** downstream ORF, *Escherichia coli*, phage regulatory switch, ribosome rescue, *trans*‐translation

## Abstract

*Escherichia coli* has multiple pathways to release nonproductive ribosome complexes stalled at the 3′ end of nonstop mRNA: tmRNA (SsrA RNA)‐mediated *trans*‐translation and stop codon‐independent termination by ArfA/RF2 or ArfB (YaeJ). The *arfA* mRNA lacks a stop codon and its expression is repressed by *trans*‐translation. Therefore, ArfA is considered to complement the ribosome rescue activity of *trans*‐translation, but the physiological situations in which ArfA is expressed have not been elucidated. Here, we found that the excision of CP4‐57 prophage adjacent to *E. coli ssrA* leads to the inactivation of tmRNA and switches the primary rescue pathway from *trans*‐translation to ArfA/RF2. This “rescue‐switching” rearranges not only the proteome landscape in *E. coli* but also the phenotype such as motility. Furthermore, among the proteins with significantly increased abundance in the ArfA^+^ cells, we found ZntR, whose mRNA is transcribed together as the upstream part of nonstop *arfA* mRNA. Repression of ZntR and reconstituted model genes depends on the translation of the downstream nonstop ORFs that trigger the *trans*‐translation‐coupled exonucleolytic degradation by polynucleotide phosphorylase (PNPase). Namely, our studies provide a novel example of *trans*‐translation‐dependent regulation and re‐define the physiological roles of prophage excision.

## Graphical abstract

In this study, we found that excision of *Escherichia coli* CP4‐57 prophage inactivates *trans*‐translation and activates the ArfA/RF2 rescue pathway back to back. This “rescue‐switching” rearranges the intercellular proteome landscape and *E. coli* phenotype in a rescue‐associated proteolysis‐dependent manner. In addition, our study also revealed that translation of the downstream nonstop ORF triggers the rescue‐associated exonucleolytic degradation and represses the expression of upstream ORFs.

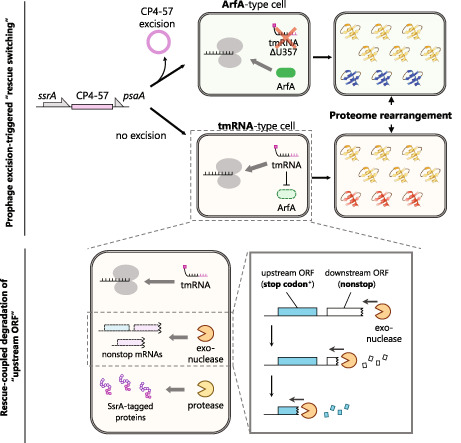

## 1 INTRODUCTION

Ribosomes translate mRNA from the start codon to the stop codon for protein synthesis. The start codon and the surrounding nucleotide sequences, such as the Shine‐Dalgarno sequence, define the entry site of the 30 S initiation complex (Rodnina, 2018). The stop codon is indispensable for the hydrolysis of the peptidyl‐tRNA within the ribosome by release factors (RF) and the following subunit dissociation from the mRNA (Rodnina, 2018). However, the translation of an aberrant mRNA lacking an in‐frame stop codon traps the ribosome at its 3′ end, due to the defect in the termination reaction. Such stop codon‐less mRNAs (nonstop mRNA) are constantly generated by mRNA degradation and premature transcription termination, and waste at least 2–4% of total translation (Ito et al., 2011; Moore & Sauer, 2005). Therefore, recycling of the ribosomes stalled on the aberrant mRNA is essential to maintain translation activity and cell viability. In fact, multiple ribosome rescue pathways are harnessed to resolve this problem in various bacterial species.

Most stalled ribosomes are rescued by tmRNA (SsrA RNA) and SmpB‐mediated *trans*‐translation (Karzai et al., 1999; Keiler et al., 1996). The *ssrA* gene is highly conserved among all bacterial genomes determined to date (Hudson & Williams, 2015; Hudson et al., 2014), implying the physiological importance of *trans*‐translation. The tmRNA molecule has both the tRNA‐like domain, which can accept alanine and the mRNA‐like domain containing an in‐frame stop codon. After the tmRNA enters the vacant A‐site of the stalled ribosome, the ribosome switches the reading frame from the nonstop mRNA to the mRNA domain of tmRNA to properly terminate the translation. This results in a truncated polypeptide translated from the nonstop mRNA (hereafter called a “nonstop polypeptide”) fused with the tmRNA‐encoded degradation tag at its C‐terminus, which promotes degradation by proteases such as ClpXP (Gottesman et al., 1998). *Trans*‐translation also induces the rapid degradation of nonstop mRNA (Yamamoto et al., 2003). Previous research using artificial constructs showed that RNase R, one of the major exonucleases in *Escherichia coli*, degrades the nonstop mRNA in a *trans*‐translation‐dependent manner (Richards et al., 2006). Thus, *trans*‐translation is a sophisticated mechanism that rescues the stalled ribosome and consequently avoids the accumulation of aberrant proteins and mRNAs.

Despite several lines of evidence supporting the importance of *trans*‐translation, many bacteria, such as *E. coli*, *Francisella tularensis*, *Bacillus subtilis*, and *Caulobacter crescentus* can survive even in the absence of the tmRNA (Keiler et al., 2000; Komine et al., 1994; Muto et al., 2001; Svetlanov et al., 2012). Some of these bacteria reportedly possess alternative ribosome rescue pathways (Chadani et al., 2010; Goralski et al., 2018; Shimokawa‐Chiba et al., 2019). ArfA (alternative ribosome rescue factor A) in *E. coli* cooperates with RF2 to terminate translation by hydrolyzing the peptidyl‐tRNA in a stop codon‐independent manner (Chadani et al., 2012; Kurita et al., 2014; Shimizu, 2012). In contrast to *trans*‐translation, the ArfA/RF2 pathway does not add a degradation tag to the released polypeptide and apparently does not recruit exonucleases to the nonstop mRNA. Therefore, nonstop polypeptides could accumulate if the stalled ribosome is released by ArfA (Chadani et al., 2011a).

ArfA expression is tightly repressed by multiple regulations (Chadani et al., 2011b; Garza‐Sánchez et al., 2011). A stem‐loop structure, which functions as both a rho‐independent terminator and a recognition site for RNase III, is encoded within the *arfA* ORF (Chadani et al., 2011b; Garza‐Sánchez et al., 2011). Therefore, the *arfA* mRNA is expressed as a nonstop mRNA in various situations and is repressed by the tmRNA‐mediated *trans*‐translation. Moreover, even if the ribosome fortuitously terminates the translation of the *arfA* ORF at the endogenous stop codon, the degradation of the full‐length ArfA protein is induced by the hydrophobic amino acid cluster at the C terminus (Chadani et al., 2011b). Therefore, ArfA is strongly repressed under the conditions where *trans*‐translation is active. In other words, ArfA likely functions as a backup mechanism in situations where the *trans*‐translation activity is somehow disturbed. Alternative ribosome rescue factors in other bacterial species are also repressed by the tmRNA, indicating that this regulation is commonly beneficial to bacterial species (Shimokawa‐Chiba et al., 2019). However, the situations in which the alternative ribosome rescue pathway is activated are poorly understood.

A recent study showed that the excision of the CP4So prophage, adjacent to the *ssrA* gene in the *Shewanella oneidensis* genome, induces a single nucleotide deletion in the *ssrA* gene and abolishes its function (Zeng et al., 2016). Such alteration of the expression/function of the host genes by prophage excision has been referred to as a “phage regulatory switch” in previous reports (Rabinovich et al., 2012; Scott et al., 2012; Tran et al., 2019). The *E. coli* K‐12 strain also has a prophage (CP4‐57) downstream from the *ssrA* gene (Kirby et al., 1994). Although CP4‐57 excision rarely occurs under normal conditions, the excision rate increases to 1–2 per 10,000 cells in specific situations, such as biofilms or long‐term cultivation (Wang et al., 2009, 2010). The excision of CP4‐57 reportedly causes the deletion of T357 in *ssrA*, which forms a wobble base pair in the acceptor stem in the tRNA‐like domain, as in *S. oneidensis* (Wang et al., 2009; Zeng et al., 2016) (Figure S1a). However, the influences of this mutation on the tmRNA activity and the activation of an alternative ribosome rescue pathway have not been elucidated.

Based on these previous findings, we hypothesized that CP4‐57 excision switches the primary ribosome rescue pathway from tmRNA‐mediated *trans*‐translation to ArfA/RF2‐mediated termination, by impairing the tmRNA activity. In this report, we found that (i) CP4‐57 excision impairs the *trans*‐translation and switches the ribosome rescue pathway from *trans*‐translation to the ArfA/RF2 pathway, and (ii) CP4‐57 excision rearranges the cellular proteome and the phenotype in “rescue‐switching”‐dependent manner. Furthermore, our analysis revealed that *zntR*, which is transcribed as a polycistronic mRNA with *arfA*, is repressed by a novel mode of *trans*‐translation‐associated regulation. The *zntR* repression is independent of proteolysis, but dependent on *trans*‐translation‐coupled exonucleolytic degradation by a polynucleotide phosphorylase (PNPase), indicating that the degradation of the *zntR* region in the mRNA is associated with that of the downstream *arfA* nonstop mRNA. Taken together with a successful reconstitution in an artificial sequence, these results indicate that the expression of the upstream ORF followed by the nonstop ORF could be widely influenced by the ribosome rescue pathways.

## 2 RESULTS

### 2.1 The excision of CP4‐57 prophage switches the ribosome rescue pathways

**FIGURE 1:**
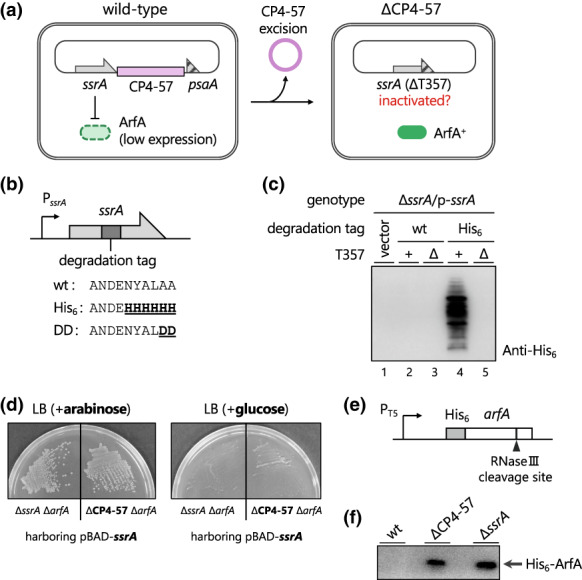
CP4‐57 prophage excision is a junction point to switch the primary ribosome rescue pathway in *Escherichia coli*. (a) Working hypothesis: the CP4‐57 excision‐induced *ssrA*ΔT357 mutation inactivates *trans*‐translation and subsequently activates the ArfA/RF2 alternative rescue pathway. (b) Schematic of the plasmids expressing tmRNA, and amino acid sequences of the degradation tags encoded within the wild‐type tmRNA and its variants, tmRNA^His^ and tmRNA^DD^. (c) Visualization of aberrant polypeptides rescued by *trans*‐translation. The *E. coli* ∆*ssrA* strain harboring pMW118 (vector control, lane 1), p‐*ssrA* (lane 2), p‐*ssrA*ΔT357 (lane 3), p‐*ssrA* ^His^ (lane 4), or p‐*ssrA* ^His^ΔT357 (lane 5) was grown in LB medium until mid‐log growth phase. The cells were then collected, extracted, fractionated by SDS‐PAGE, and analyzed by western blotting using an anti‐His_6_ antibody. (d) Synthetic lethal phenotype of ΔCP4‐57 and Δ*arfA* mutation. *E. coli* Δ*ssrA* Δ*arfA* and the ΔCP4‐57 Δ*arfA* strain harboring pBAD‐*ssrA* were grown on LB plates containing 0.2% arabinose (left) or 0.4% glucose (right) at 37°C overnight. (e) Schematic of the plasmid {pCH200 (Chadani et al., 2010)} expressing N‐terminally His_6_‐tagged ArfA. The *arfA* ORF and the His_6_‐tag sequence are shown by open and filled boxes, respectively. The IPTG‐inducible LacO‐T5 promoter (P_T5_) and the RNase III cleavage site are also shown. (f) Expression profile of His_6_‐ArfA in wild‐type, ΔCP4‐57 and Δ*ssrA* strains. Each strain harboring pCH200 was cultured in LB medium until the OD_660_ reached 0.2–0.3. The expression of ArfA was then induced for 2 h in the presence of 500 μM IPTG. The extracts of each strain were analyzed by western blotting using an anti‐His_6_ antibody.

Based on several previous reports, we hypothesized that the CP4‐57 excision‐triggered *ssrA*ΔT357 mutation inactivates *trans*‐translation and consequently switches the ribosome rescue pathway to the alternative ArfA/RF2 pathway (Figure 1a). To evaluate the *trans*‐translation activity of the tmRNAΔU357 mutant, we examined whether tmRNA‐tagged polypeptides are accumulated in cells. Since tmRNA‐tagged polypeptides are rapidly degraded, we used tmRNA^His^, a tmRNA variant encoding a proteolysis‐resistant hexahistidine tag (Roche & Sauer, 2001) (Figure 1b). Cellular extracts of *ssrA* deletion mutants expressing tmRNA^His^ or tmRNA^His^ΔU357 were analyzed by western blotting, using an anti‐His_6_ antibody (Figure 1c). In the presence of tmRNA^His^, numerous polypeptides with various molecular weights were detected, indicating the *trans*‐translation reaction by the tmRNA^His^ mutant, as reported previously (Roche & Sauer, 2001). In contrast, the expression of tmRNA^His^ΔU357 did not produce any polypeptide containing the His_6_‐tag, indicating that the ΔU357 mutation disrupts the *trans*‐translation activity of the *E. coli* tmRNA. Note that the possibility of tmRNA inactivation by prophage excision has been suggested for other γ‐proteobacteria that possess a homolog of *arfA*, but has not been directly evaluated (Liu et al., 2019; Rajanna et al., 2003; Wang et al., 2009; Williams, 2003; Zeng et al., 2016) (Table 1). Accordingly, we introduced the mutations found in those bacteria into the *E. coli* tmRNA, and confirmed the robust inactivation of the *trans*‐translation activity, which was similar to the ΔU357 mutation (Figure S1b lanes 6 and 7).

**TABLE 1:** Prophage excision‐induced mutations in *ssrA* gene among bacterial species

Bacterial species	Prophage	Integrase type	Mutation at the 3′ end of *ssrA*	Reference
*Escherichia coli* K‐12	CP4‐57	P4 integrase	CGCCAGC‐CCACCA	Wang et al. (2009)
*Shewanella oneidensis* MR‐1	CP4So	P4 integrase	CGCCAGC‐CCACCA	Zeng et al. (2016)
*Vibrio cholerae* N16961	VPIΦ	P4 integrase	CCCCAGC‐CCACCA	Rajanna et al. (2003); Williams (2003)
*Salmonella enterica* serovar Typhimurium LT2	Stm27X	P4 integrase	CGCCAGC‐‐‐‐‐CA	Williams (2003)
*Salmonella enterica* serovar Typhi TY2	Stit11X	P4 integrase	CGCCAGC‐CCACCA	Williams (2003)
*Yersinia pestis* CO92	Ype11X	P4 integrase	CCCCAGC‐‐‐‐CCA	Williams (2003)
*Shewanella putrefaciens*	‐	P2 integrase	CGCCAGC‐CCACCA	Liu et al. (2019)
*Escherichia coli* RS218	Eco48X	CTXΦ integrase	CGCCAGC‐‐‐‐‐CA	Williams (2003)
*Escherichia coli* O157:H7 EDL933	Oi108	unclassified	CGCCAGC‐‐‐‐‐CA	Williams (2003)
*Salmonella Typhimurium* DT204	SopEΦ	P2 integrase	CGCCAGCTCCACCA	Pelludat et al. (2003)
*Salmonella enterica* serovar Typhimurium LT2	Fels‐2	P2 integrase	CGCCAGCTCCACCA	Williams (2003)
*Salmonella enterica* serovar Paratyphi A	SpaX	P2 integrase	CGCCAGCTCCACCA	Williams (2003)
*Escherichia coli* CFT073	Ecoc48X	CTXΦ integrase	CGCCAGCTCCACCA	Williams (2003)

We previously reported that *E. coli* cells lacking both tmRNA and ArfA are unable to grow due to the accumulation of nonstop translation complexes (Chadani et al., 2010). Therefore, we examined whether the deletion of *arfA* and the CP4‐57 excision (∆CP4‐57 *ssrA*∆T357) also elicit a synthetic lethal phenotype. The CP4‐57 excision was induced by the overexpression of the AlpA protein, as described previously (Wang et al., 2009, 2010). The *arfA* gene was then deleted in the presence of pBAD‐*ssrA*, which produces the tmRNA in response to arabinose (Chadani et al., 2010). As expected, the ∆CP4‐57 ∆*arfA* strain, as well as the ∆*ssrA* ∆*arfA* double mutant, grew only in the presence of arabinose (Chadani et al., 2010) (Figure 1d). This result further confirmed that tmRNAΔU357 impairs the *trans*‐translation activity.

Finally, we investigated whether the ribosome rescue pathway is actually switched from *trans*‐translation to the ArfA/RF2 pathway in the ΔCP4‐57 strain. We expressed *arfA* encoding the N‐terminal His_6_‐tag and the stem‐loop for RNase III cleavage in *E. coli* strains (Figure 1e,f). The His_6_‐ArfA protein did not accumulate in the wild‐type strain, due to the tight repression by *trans*‐translation (Chadani et al., 2011b; Garza‐Sánchez et al., 2011). In contrast, the ArfA protein was expressed in the ΔCP4‐57 strain and the ∆*ssrA* mutant. This result clearly demonstrated that the ArfA/RF2 pathway is the primary system to rescue the nonstop translation complex in the ΔCP4‐57 strain. From these results, we concluded that the excision of *E. coli* CP4‐57 functions as a phage regulatory switch to convert the primary ribosome rescue pathway from tmRNA‐mediated *trans*‐translation to the ArfA/RF2 system.

### 2.2 CP4‐57 excision rearranges the *E. coli* proteome by switching the ribosome rescue pathways

**FIGURE 2:**
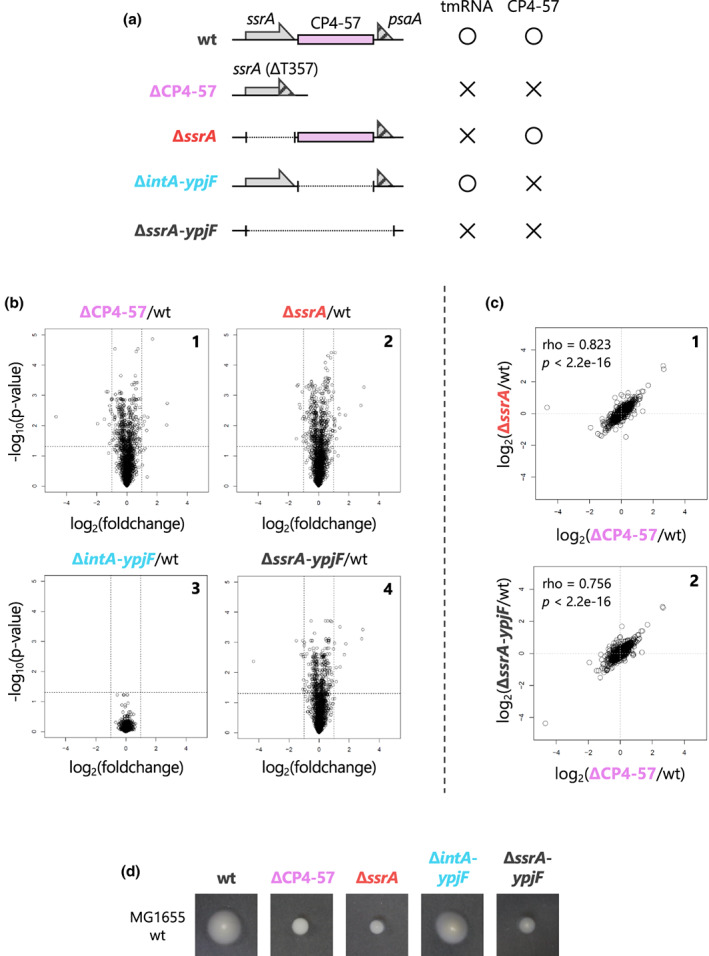
CP4‐57 excision rearranges the proteome landscape in *Escherichia coli* cells by switching the primary ribosome rescue mechanism. (a) Schematic drawing of the genomic structure of the *ssrA* ‐ CP4‐57 locus in the *E. coli* BW25113 strain (wild type: wt) and its derivatives, ΔCP4‐57, ∆*ssrA*, Δ*intA*‐*ypjF* (lacking the entire sequence of CP4‐57 prophage region while retaining intact *ssrA*) and ∆*ssrA*‐*ypjF*. *E. coli* cells were grown in LB medium until mid‐log phase (OD_660_ ~ 0.5) and collected for the SWATH analysis (See “Experimental procedure” for details). (b) The degrees of proteomic rearrangement between BW25113 (wt) and its derivatives (∆CP4‐57: panel 1, ∆*ssrA*: panel 2, ∆*intA‐ypjF*: panel 3, ∆*ssrA*‐*ypjF*: panel 4) are represented by volcano plots. Each dot represents the fold change and the *p*‐value of each protein identified by the SWATH‐MS analysis plotted according to its relative abundance ratio (log_2_ fold change) on the horizontal axis and *p*‐value on the vertical axis. The lines indicate a *p*‐value of 0.05 and twofold change. (c) Two‐dimensional plots of the fold change values in the *E. coli* BW25113 mutant are indicated below and on the side of the graph. Each plot is represented with Spearman's rho and *p*‐values calculated by Spearman's rank correlation tests. (d) Swimming motility of MG1655 wild‐type strain and its derivatives, the ΔCP4‐57, Δ*ssrA*, Δ*intA*‐*ypjF*, and ∆*ssrA*‐*ypjF* mutants. Colonies were inoculated onto semisolid agar plates and incubated at 30°C for 20 h.

Both tmRNA and ArfA/RF2 rescue stalled ribosomes; however, only the former pathway induces the degradation of both the released polypeptide and the nonstop mRNA (Chadani et al., 2010, 2011a). Therefore, we assumed that the CP4‐57 excision apparently shuts off the clearance mechanism for nonstop mRNAs and their translational products. This would result in the accumulation of nonstop polypeptides and thus rearrange the proteome landscape. To validate this possibility, we performed a quantitative proteomics analysis by using the SWATH‐MS acquisition method (Gillet et al., 2012). We compared the *E. coli* BW25113 standard strain (wild‐type) and its derivatives, ΔCP4‐57, ∆*ssrA*, Δ*intA*‐*ypjF* (lacking the genes within the CP4‐57 prophage region while retaining *ssrA*), and ∆*ssrA*‐*ypjF*, as summarized in Figure 2a. Each strain was cultivated in LB broth until the mid‐log phase and then processed for the comparative analyses. The fold change values of each protein were calculated as the division of the MS intensity in the mutant by that in the wild‐type standard strain. Volcano plots revealed the dynamic rearrangement of the proteome landscape in the ∆CP4‐57, ∆*ssrA*, and ∆*ssrA*‐*ypjF* mutants (Figure 2b). Interestingly, the regulons of the heat shock transcriptional regulator, σ32, were upregulated in the tmRNA‐deficient cells (Nonaka et al., 2006) (Figure S2a). This result is consistent with the previous report that the heat shock response is caused by the accumulation of aberrant polypeptides in the ∆*ssrA* mutant (Munavar et al., 2005). In contrast to the other mutations, the Δ*intA*‐*ypjF* deletion did not affect the proteome landscape under our test conditions.

Next, we compared the proteomic rearrangements in each mutant. Two‐dimensional plots revealed that the proteomic rearrangement by the ΔCP4‐57 mutation correlated well with those of the ∆*ssrA* and ∆*ssrA*‐*ypjF* mutants (Figure 2c), but not with the Δ*intA*‐*ypjF* mutant (Figure S2b). These tendencies were also observed in *E. coli* cells in the stationary growth phase and those grown in a defined minimal medium (Figure S2c). These results indicated that the proteomic rearrangement depends on the inactivation of tmRNA, but not the loss of prophage‐related genes in the CP4‐57 region. In addition, the expression of tmRNAΔU357 in the ∆*ssrA* mutant had no effect, excluding the possibility that the tmRNAΔU357 molecule gains a novel biological function to rearrange the proteome (Figure S2d). We then concluded that the proteomic rearrangement by the ΔCP4‐57 depends on the tmRNA inactivation by the U357 deletion.

We also examined whether the phenotypic alteration by the CP4‐57 excision also depends on the switching of ribosome rescue pathways. Wang and colleagues reported that the CP4‐57 excision affects several phenotypes, such as motility (Wang et al., 2009). Therefore, we introduced the mutations shown in Figure 2a into the highly motile *E. coli* MG1655 strain, because the BW25113 strain, used in the previous study (Wang et al., 2009), is motility‐impaired (Wood et al., 2006) (Figure S2e). The proteomic rearrangements in MG1655 and its derivatives generated almost identical results to those of the BW25113 strains (Figure S2f). All of the ΔCP4‐57, ∆*ssrA*, and ∆*ssrA*‐*ypjF* mutants that lacked the *trans*‐translation activity swam poorly (Figure 2d), consistent with the previous study by Komine and colleagues, who reported the weakened motility phenotype of the ∆*ssrA* mutant (Komine et al., 1994), supporting our assumption. From these results, we concluded that the CP4‐57 excision rearranges the proteome landscape and changes the motility phenotype by switching the ribosome rescue pathways.

### 2.3 The absence of ribosome rescue‐associated proteolysis of nonstop polypeptides triggers a dynamic rearrangement of the proteome

**FIGURE 3:**
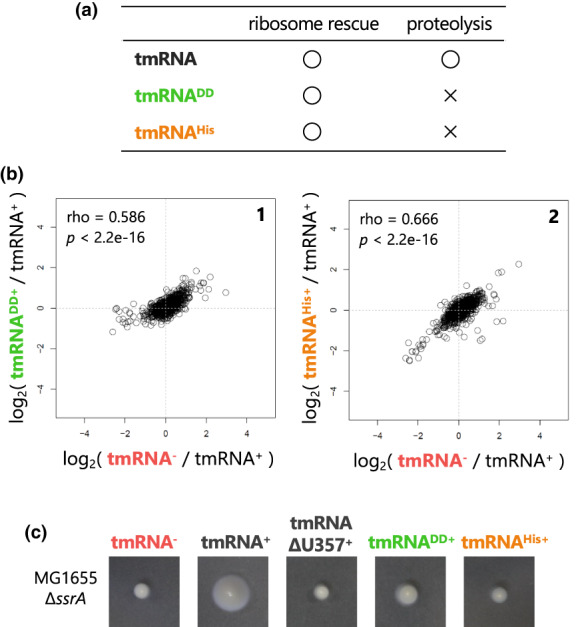
The absence of ribosome rescue‐associated proteolysis of a nonstop polypeptide triggers the rearrangement of the cellular proteome. (a) Functional comparison of wild‐type tmRNA and degradation tag mutants (tmRNA^DD^ and tmRNA^His^) that rescue the stalled ribosome but poorly induce the proteolysis of the released polypeptide (Gottesman et al., 1998; Herman et al., 1998; Keiler et al., 1996; Roche & Sauer, 2001). The MG1655 Δ*ssrA* strain harboring pMW118 (vector control) and its derivatives expressing the tmRNA variant were grown and analyzed as shown in Figure 2. (b) Two‐dimensional plots of the fold change values in each *Escherichia coli* strain expressing tmRNA or proteolysis‐deficient mutants. Each plot is represented with Spearman's rho and *p*‐values calculated by Spearman's rank correlation tests. (c) Swimming motility of the MG1655Δ*ssrA* strains harboring pMW118 and its derivatives, p‐*ssrA*, p‐*ssrA*ΔT357, p‐*ssrA* ^DD^. and p‐s*srA* ^His^. Colonies were inoculated onto semisolid agar plates and incubated at 30°C for 20 h.

Both *trans*‐translation and alternative rescue by ArfA commonly rescue stalled ribosomes, but only the former pathway induces the degradation of nonstop polypeptides. Accordingly, we assumed that the major determinant of the proteomic rearrangement by the CP4‐57 excision would be the presence or absence of the degradation mechanism for the nonstop polypeptides between the two rescue pathways. To assess this, we again performed the SWATH‐MS analysis for *E. coli* expressing the tmRNA^DD^ or tmRNA^His^ variant, which rescues the stalled ribosome but poorly induces the proteolysis of nonstop polypeptides (Gottesman et al., 1998; Herman et al., 1998; Keiler et al., 1996; Roche & Sauer, 2001) (Figures 1b and 3a). According to our hypothesis, we expected that the proteomic rearrangement in the tmRNA^DD^ or tmRNA^His^ strain would share some similarity with that in the ∆*ssrA* mutant.

*E. coli* expressing the tmRNA^DD^ or tmRNA^His^ mutant showed a certain degree of proteomic rearrangement, similar to that of the ∆*ssrA* mutant, relative to the wild‐type tmRNA‐expressing strain (Figure S3a). Furthermore, the proteomic rearrangements in these tmRNA mutants correlated well with that of the ∆*ssrA* mutant (Figure 3b). Actually, a moderate heat shock‐like response was commonly induced in these proteolysis‐deficient tmRNA‐expressing cells and the ∆*ssrA* cells (Figure S3b). From these results, we concluded that the presence or absence of ribosome rescue‐associated proteolysis activity is the major determinant of the proteomic rearrangement by the CP4‐57 excision. The weakened motility phenotype of *E. coli* lacking the rescue‐dependent proteolysis also supports this notion (Okan et al., 2006) (Figure 3c).

### 2.4 ZntR expression is repressed in a *trans*‐translation‐dependent manner, but is independent of the degradation tag‐induced proteolysis

**FIGURE 4:**
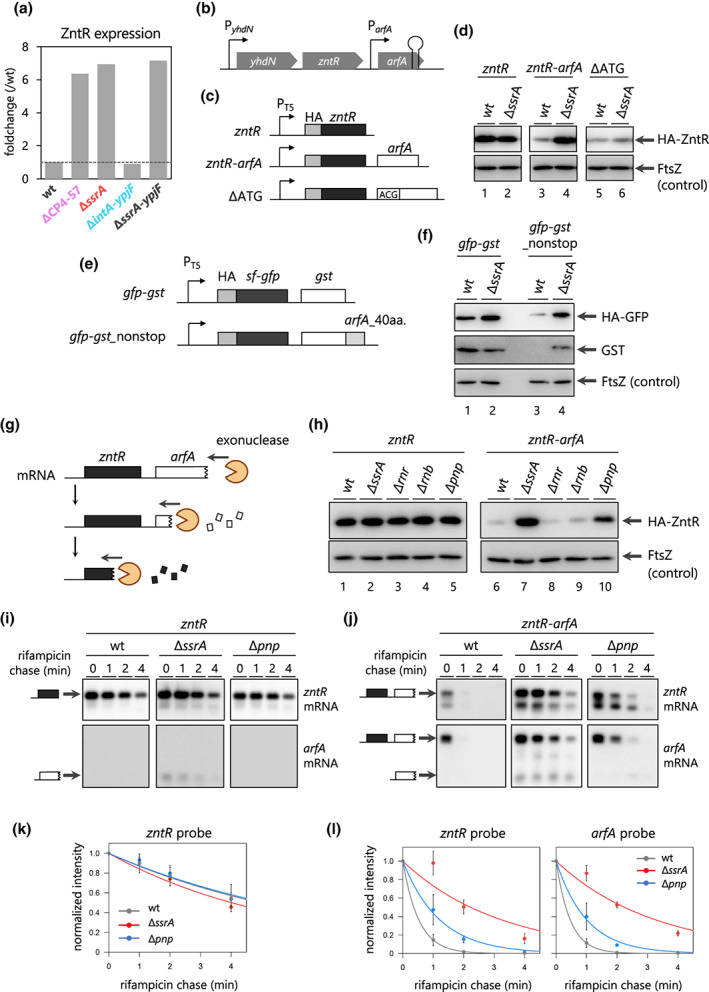
*zntR* mRNA is involved in the *trans*‐translation‐dependent degradation of *arfA* nonstop mRNA by the PNPase exonuclease. (a) Expression level of ZntR quantified by the SWATH‐MS analyses in Figure 2. The dashed line indicates a fold change value of 1. (b) Genetic structure of the *yhdN*‐*zntR‐arfA* locus. The transcription promoters of *yhdN* (P_*yhdN*_) and *arfA* mRNA (P_*arfA*_) and the intrinsic transcriptional terminator within the *arfA* ORF are schematically indicated. (c) Schematic representations of the N‐terminally HA‐tagged *zntR* and *zntR*‐*arfA*. The initiation codon of *arfA* AUG was changed to ACG to deplete the translation of nonstop *arfA* mRNA (∆ATG). (d) Expression of HA‐ZntR in the absence of tmRNA. The BW25113 (wt) and Δ*ssrA* strains harboring pOH012 (*zntR*, lanes 1 and 2), pOH008 (*zntR*‐*arfA*, lanes 3 and 4), or pOH019 (ΔATG, lanes 5 and 6) were grown in LB medium until the OD_660_ reached 0.2–0.3. IPTG (100 μM) was then added to induce the expression of *zntR*, and cells were grown until the OD_660_ doubled. The cellular extracts were analyzed by western blotting using an anti‐HA‐tag antibody. (e) Schematic representation of sfGFP‐GST constructs. The entire sequences of the *zntR* and *arfA* ORFs are replaced by sfGFP (Superfolder GFP) and GST (glutathione‐S transferase), respectively. The region encoding the C‐terminal 40 amino acids of *arfA* was fused with the *gst* ORF to post‐transcriptionally remove its stop codon (*gst*_nonstop). (f) Expression of sfGFP and GST in BW25113 (wt) or the Δ*ssrA* strain harboring pOH074 (*gfp*‐*gst*, lanes 1 and 2) or pOH075 (*gfp*‐*gst*_nonstop, lanes 3 and 4). Cellular extracts were prepared as shown in Figure 4d and analyzed by western blotting using an anti‐GFP tag antibody (upper panel), an anti‐GST tag antibody (middle panel), and an anti‐FtsZ tag antibody (lower panel). (g) A working hypothesis for tmRNA‐dependent ZntR repression: the *zntR* mRNA is involved in the exonucleolytic degradation of the *arfA* nonstop mRNA. (h) Expression of HA‐ZntR in the mutants lacking one of the major exonucleases. BW25113 (wt) and its derivatives, Δ*ssrA*, Δ*rnr*, Δ*rnb*, and Δ*pnp* strains harboring pOH090 (*zntR*, lanes 1–5) or pOH089 (*zntR*‐*arfA*, lanes 6–10) were analyzed as in Figure 4d. (i) Stability of the *zntR* monocistronic mRNA. Schematic labels indicate the *zntR* mRNA (filled box) and the nonstop *arfA* mRNA (open box). The wild‐type, Δ*ssrA* strain and ∆*pnp* strain harboring pOH090 (*zntR*) were grown in LB medium until mid‐log phase. The expression of *zntR* mRNA was then induced by 1 mM IPTG for 15 min. At this point, 200 μg/ml of rifampicin was added to the culture. The total RNA was extracted from the cells at 0, 1, 2, and 4 min after rifampicin treatment. Total RNAs were analyzed by northern blotting using an anti‐*zntR* mRNA probe (upper panel) and an anti‐*arfA* mRNA probe (lower panel). (j) Stability of the *zntR*‐*arfA* polycistronic mRNA. Schematic labels indicate the *zntR*‐*arfA* mRNA and the *arfA* mRNA. Wild‐type, Δ*ssrA* and Δ*pnp* strains harboring pOH089 (*zntR*‐*arfA*) were grown in LB medium until mid‐log phase. The expression of the *zntR*‐*arfA* mRNA was then induced by 1 mM IPTG for 15 min. At this point, 200 μg/ml of rifampicin was added to the culture. Total RNAs were prepared as Figure 4i and analyzed by northern blotting using an anti‐*zntR* mRNA probe (upper panel) and an anti‐*arfA* mRNA probe (lower panel). (k) The quantified intensity of the *zntR* mRNA is shown in Figure 4i. Error bars represent the SD values of three biological replicates. (l) The quantified intensity of the *zntR*‐*arfA* mRNA is shown in Figure 4j. Error bars represent the SD values of three biological replicates.

In the course of the MS analyses, we noticed that the deficiency of the tmRNA reproducibly increased the expression of ZntR, a transcriptional regulator for the ZntA transporter that exports divalent metal ions such as Zn^2+^ (Figure 4a). Interestingly, *zntR* is located just upstream of *arfA*, which is expressed as a nonstop mRNA (Figure 4b). Due to the absence of a typical rho‐independent terminator in the intergenic region between *zntR* and *arfA* (Figure S4a), these genes are likely to be transcribed as a polycistronic mRNA. Accordingly, we hypothesized that the tmRNA‐dependent repression of *zntR* is somehow coupled with the translation of the *arfA* nonstop mRNA. To assess this, the expression levels of ZntR with or without the following *arfA* ORF were examined by immunoblotting, using an N‐terminal HA‐tagged ZntR (Figure 4c). When *zntR* was expressed as a monocistronic mRNA, the tmRNA had no impact on the expression of the ZntR protein (Figure 4d lanes 1–2), confirming that ZntR is not a direct substrate of *trans*‐translation. Disruption of the *arfA* promoter within the *zntR* ORF had no effect on the regulation of ZntR, excluding the possibility of premature transcription termination as in the case of *lacI* (Abo et al., 2000) (Figure S4b). In the presence of tmRNA, the ZntR protein was poorly expressed from the *zntR‐arfA* polycistronic mRNA (Figure 4d lanes 3–4). In addition, a disruption of the initiation codon for the *arfA* ORF abolished the tmRNA‐dependent repression of ZntR (Figure 4d lanes 5–6). From these results, we concluded that the translation of the nonstop *arfA* ORF induces the repression of ZntR in a *trans*‐translation‐dependent manner.

### 2.5 Reconstitution of *trans*‐translation‐dependent repression of the upstream ORF in artificial sequences

The results described above indicated that the repression of ZntR is independent of the tmRNA‐driven proteolysis of ZntR itself. To verify this further, we replaced the *zntR* and *arfA* ORFs with sfGFP (Superfolder GFP) and GST (glutathione S‐transferase), respectively (Figure 4e). The region encoding the C‐terminal 40 amino acids of *arfA* was translationally fused with the *gst* ORF to post‐transcriptionally remove its stop codon (*gst*_nonstop). Translation of the *gfp*‐*gst* polycistronic mRNA produced the sfGFP and GST proteins in a constant ratio, irrespective of the tmRNA (Figure 4f lanes 1–2). In contrast, the expression of the sfGFP protein from the *gfp‐gst*_nonstop mRNA, as well as that of the downstream GST, was significantly repressed in the presence of the tmRNA (Figure 4f lanes 3–4). This result is quite consistent with the case of the *zntR*‐*arfA* nonstop mRNA. These results indicate that the repression of ZntR is independent of its amino acid sequence and dependent on the context of the ORF followed by the nonstop ORF.

### 2.6 *zntR* mRNA is involved in *trans*‐translation‐dependent degradation of *arfA* nonstop mRNA by exonuclease PNPase

*Trans*‐translation stimulates the exonuclease degradation of nonstop mRNA (Richards et al., 2006). Therefore, we hypothesized that ZntR expression is repressed at the mRNA level; namely, tmRNA‐dependent exonucleolytic degradation of *zntR‐arfA* mRNA is responsible for the ZntR repression by tmRNA. (Figure 4g). Actually, the insertion of an artificial stem‐loop into the intergenic region between *zntR* and *arfA* abolished the tmRNA‐dependent repression of ZntR, supporting this assumption (Figure S4c). We evaluated the expression of ZntR in *E. coli* strains lacking one of the three major 3′ → 5′ exonucleases in *E. coli*. The monocistronic *zntR* mRNA was translated into the ZntR protein at almost identical levels in the wild‐type and the ∆*ssrA* and exonuclease‐lacking mutants (Figure 4h lanes 1–5). In contrast, the *zntR*‐*arfA* polycistronic mRNA allowed the synthesis of ZntR only in the ∆*ssrA* and ∆*pnp* mutants (Figure 4h lanes 7, 10). This result indicated that PNPase, encoded by the *pnp* ORF, degrades the *zntR*‐*arfA* mRNA in a *trans*‐translation‐dependent manner. PNPase is unable to degrade stable stem‐loop structures in mRNA (Spickler & Mackie, 2000), consistent with the influence of the stem‐loop insertion (Figure S4c).

To obtain direct evidence, we next evaluated the stability of the *zntR*‐*arfA* mRNA in the *E. coli* ∆*pnp* mutant. We stopped the synthesis of nascent mRNAs by the addition of rifampicin and extracted cellular RNA at the indicated time points. The mRNA molecules containing the *zntR* or *arfA* sequence were then individually probed by northern blotting. The monocistronic *zntR* mRNA was equivalently stable in all *E. coli* strains tested, again confirming that the *zntR* mRNA itself is not affected by *trans*‐translation or PNPase (Figure 4i,k). In contrast, the *zntR*‐*arfA* polycistronic mRNA was extremely unstable in the wild‐type strain, but significantly stabilized in the ∆*ssrA* and ∆*pnp* mutants (Figure 4j,l). These results indicated that *trans*‐translation triggers the PNPase‐dependent degradation of the *zntR*‐*arfA* mRNA so that the expression of ZntR is repressed in the presence of the tmRNA. Consistent with this notion, we reanalyzed the proteomic datasets of the tmRNA variants (Figure 3) and found that the tmRNA^DD^ mutant also repressed the ZntR expression (Figure S4d). The tmRNA^DD^ variant is defective in the proteolysis of nonstop polypeptides, but efficiently promotes the degradation of nonstop mRNA as well as the wild‐type tmRNA (Yamamoto et al., 2003). From these results, we concluded that *trans*‐translation regulates the ZntR expression in a proteolysis‐independent and exonucleolytic degradation‐dependent manner.

## 3 DISCUSSION

Previous studies of ArfA, the tmRNA‐dependent tight repression, and the cooperative action with RF2 to release stalled ribosomes have been performed from a variety of perspectives, including biochemical and structural analyses (Chadani et al., 2011b; Garza‐Sánchez et al., 2011; Huter et al., 2017; James et al., 2016; Kurita et al., 2014; Ma et al., 2017; Zeng et al., 2017). However, the situations in which the ArfA/RF2 pathway is activated to compensate for the ribosome rescue function, and the impact on the cellular proteome when the ribosomal rescue pathway is actually switched to the alternative rescue pathway, have not been elucidated.

**FIGURE 5:**
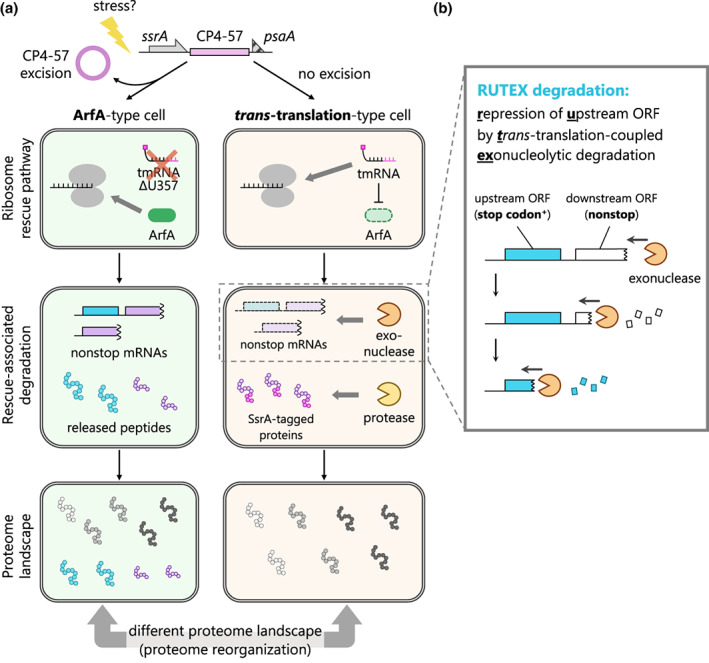
Proposed mechanism of the *Escherichia coli* proteome rearrangement by the CP4‐57 excision. (a) Excision of the CP4‐57 prophage from the *E. coli* genome is triggered by various environmental stresses. This DNA rearrangement also induces a single nucleotide deletion in *E. coli ssrA*, which impairs the *trans*‐translation activity and subsequently activates the ArfA/RF2 ribosome rescue pathway back‐to‐back. This switching results in the accumulation of nonstop polypeptides that are usually repressed by *trans*‐translation‐coupled proteolytic and nucleolytic degradation, thus reorganizing the proteome landscape. (b) Schematic of the repression of upstream ORF by *trans*‐translation‐coupled exonucleolytic (RUTEX) degradation.

In this study, we demonstrated for the first time that the excision of the CP4‐57 prophage, located downstream of the *E. coli ssrA* gene, functions as a phage regulatory switch to inactivate the tmRNA and to simultaneously activate the ArfA/RF2 ribosome rescue system (Figure 1). The excision of CP4‐57 deletes the U357 residue of tmRNA, which forms a G･U wobble base pair in the acceptor stem of tmRNA (Wang et al., 2009). Biochemical and structural analyses of the wobble base pair have shown its importance for the recognition of alanyl‐tRNA synthetase (Giegé et al., 1998; Hou & Schimmel, 1988; McClain & Foss, 1988; Naganuma et al., 2014), indicating that tmRNAΔU357 has an aminoacylation defect. This inactivation of the tmRNA rearranged the intracellular proteome of *E. coli* and also altered the phenotype (Figure 2). Furthermore, the similarity between the proteomic rearrangements among ∆*ssrA* and the proteolysis‐deficient tmRNA mutants indicates that the accumulation of the nonstop polypeptide is the major determinant for the reorganization of the *E. coli* proteome (Figure 3, summarized in Figure 5a).

In addition to *S. oneidensis, E. coli* and other γ‐proteobacteria possess phage regulatory switches that shut off the function of the tmRNA (Table 1 and Figure S1b). Since all bacterial species with the prophage‐dependent tmRNA‐inactivation mechanism also have *arfA* genes, the “rescue‐switching” would occur, as in *E. coli*. However, the excision of other *ssrA*‐adjacent prophages does not always modify the sequence of the *ssrA* gene, even though their host cells possess *arfA* (e.g., *Salmonella Typhimurium* DT204 and *E. coli* CFT073, Table 1) (Pelludat et al., 2003; Williams, 2003). Therefore, the phage regulatory switches that regulate the ribosome rescue functions among different bacterial species were probably acquired independently after the phage integration.

At first glance, the inactivation of *trans*‐translation is merely a defective event for the survival of bacterial cells, as reported in previous studies (Abo et al., 2002; Hobbs et al., 2010; Komine et al., 1994; Oh & Apirion, 1991). However, the excision of CP4So in *S. oneidensis* or CP4‐57 in *E. coli* is induced by various environmental stresses and contributes to survival in certain situations (Wang et al., 2009; Zeng et al., 2016). In this context, it is noteworthy that the excisions of CP4So and CP4‐57 contributed to biofilm formation through a stochastic differentiation of the bacterial subpopulations. Taken together with the previous report that biofilm formation requires a transition from a motile to a non‐motile state (Guttenplan & Kearns, 2013), the non‐motile ΔCP4‐57 cells would form the early stage of the biofilm as the subpopulation appeared from the motile wild‐type cells. In general, the excision of prophages including CP4‐57 in *E. coli* is induced stochastically in a small part of the bacterial population under certain stress conditions, consistent with this assumption (Wang et al., 2010). Nevertheless, as environmental conditions improve, the wild‐type *ssrA* ^+^ strain would predominate and the ∆CP4‐57 strain will disappear. However, the determinants for the frequency of the prophage excision and the overall landscape of the environmental stresses that trigger the excision are not fully understood. In addition, whether the excision/integration of CP4‐57 is reversible is important for the mechanism by which prophage excision in general serves as a regulatory switch of the rescue pathway for physiological purposes other than biofilm formation. Further analyses will be required to elucidate the biological significance of prophage excision‐associated rescue‐switching.

Using proteomics analyses as a starting point, we revealed that the expression of ZntR is repressed by PNPase‐dependent degradation, which is independent of the sequence features of *zntR* but dependent on the following nonstop *arfA* ORF (Figure 4). To our knowledge, previous related studies mainly focused on the translation of the stop codon‐less ORF itself, and thus the influence of *trans*‐translation on the neighboring genes such as *zntR* would have been overlooked. Accordingly, our findings on *zntR* expand the repertoire of *trans*‐translation‐dependent gene regulation and differ from the canonical substrates of tmRNA in the following points: (i) *zntR* (and maybe other genes) is regulated through *trans*‐translation even though its mRNA maintains the stop codon and (ii) the expression of ZntR is repressed by mRNA degradation and is independent of the degradation tag‐dependent proteolysis. Here, we propose to define this repression of upstream ORF by *trans*‐translation‐coupled exonucleolytic degradation as RUTEX degradation (Figure 5b).

From another viewpoint, RUTEX degradation determines the stability of the target mRNA by the translation status of the 3′ downstream region, irrespective of the translation of its own ORF. Expanding this notion, we assume that RUTEX degradation can also regulate an apparent monocistronic mRNA carrying an intact ORF. For example, translation of the 3’ UTR by stop codon readthrough or frameshifting may induce *trans*‐translation at the regular 3′ end of the mRNA and the following mRNA degradation by exonuclease(s). Thus, it is possible that RUTEX degradation extends the role of *trans*‐translation beyond the degradation of aberrant products. RUTEX degradation might define the limit of translation frequency per each mRNA in a cooperative action with the stochastic occurrence of noncanonical translation, such as stop codon readthrough and frameshifting at the C‐terminus of the ORF. Our assumption contrasts with the well‐studied concept that the translation of upstream ORFs (uORFs, or leader peptides) determines the translation frequency of the downstream ORF or mRNA expression (Dever et al., 2020). Interestingly, several groups reported that the translation of the 3’ UTR also regulates the translation frequency of the main ORF in eukaryotes (Yordanova et al., 2018). RUTEX degradation might play a similar role in prokaryotes.

We found that the *zntR*‐*arfA* mRNA is primarily degraded by PNPase (Figure 4h–l). Herzel and colleagues recently reported that the amount of the *arfA* transcript increases in a Δ*pnp* strain (Herzel et al., 2022), in agreement with our results. However, the contribution of PNPase disagrees with a previous finding that RNase R is responsible for the degradation of nonstop mRNA (Richards et al., 2006). We expect that one of the determinants for this divergence is the presence or absence of the secondary structure at the 3′ end of the mRNA. In detail, the expression of the artificial lambda cI nonstop mRNA is achieved by the insertion of the *trpA* rho‐independent terminator within the cI ORF (Richards et al., 2006), whereas the deletion of the *arfA* stop codon is dependent on the endonucleolytic cleavage by RNase III, thus removing the stem‐loop structure (Chadani et al., 2011b; Garza‐Sánchez et al., 2011). PNPase is unable to degrade mRNAs that form a stable stem‐loop (Spickler & Mackie, 2000), while in contrast, RNase R exerts its exonuclease activity regardless of the stem‐loop (Awano et al., 2010; Cheng & Deutscher, 2005). According to these previous findings and our results, the degradation of nonstop mRNAs is not likely to be uniform, as previously considered. Rather, we assume that exonucleases such as RNase R, PNPase, and RNase II cooperatively degrade a large variety of nonstop mRNAs, and the preference for each substrate depends on the shape of the 3′ end of each nonstop mRNA. The *zntR*‐*arfA* mRNA was relatively stable in the ∆*pnp* strain but more stable in the ∆*ssrA* strain, probably reflecting the contribution of other exonuclease(s) in the absence of PNPase (Figure 4l). The existence of multiple exonucleases in bacterial species would contribute to the robust degradation of the aberrant mRNAs. However, the reason why the expression of ZntR is associated with *trans*‐translation is unknown. The *zntR* genes among the enterobacteria often form an operon structure with *arfA*, suggesting a potential benefit via the rescue‐associated expression regulation of ZntR (Figure S5). The ZntR protein up‐regulates the ZntA Zn^2+^ exporting transporter (Binet & Poole, 2000; Brocklehurst et al., 1999). Interestingly, several Zn^2+^‐containing ribosomal proteins such as bL31A (RpmE) are replaced with their Zn^2+^‐free orthologs (bL31B: YkgM) under Zn^2+^‐depleted conditions (Natori et al., 2007). Such a replacement of the ribosomal components may alter the function of the ribosome itself, as in the concept of “specialized ribosome” in eukaryotes (Gay et al., 2022). Actually, Lilleorg et al reported that the ribosome equipped with bL31A shows some differences from that with bL31B (Lilleorg et al., 2020). Rescue‐dependent Zn^2+^‐homeostasis regulation might associate with the alternation of the translational machinery to adapt to the stress conditions in which *trans*‐translation activity is insufficient or the CP4‐57 excision is induced. However, neither expression of ZntA nor Zn^2+^‐containing ribosomal proteins was altered under the conditions we tested (Table S6). Further analyses are required to elucidate the detailed mechanism of the exonuclease recruitment and to clarify the overall picture and biological significance of the rescue‐associated mRNA quality control in bacterial species.

## 4 EXPERIMENTAL PROCEDURES

### 4.1 *E. coli* strains, plasmids, and primers

*E. coli* strains, plasmids, and primers used in this study are listed in Tables [Link], [Link], and S3, respectively. Phage P1‐mediated transduction was used to introduce the *arfA*, *rnr*, *rnb*, and *pnp* mutations from the Keio collection JW3253, JW5741, JW1279, and JW5851, respectively (Baba et al., 2006). The ∆*ssrA*, ∆*intA*‐*ypjF*, and ∆*ssrA*‐*ypjF* mutations were introduced by homologous recombination (Datsenko & Wanner, 2000) with a chloramphenicol resistance cassette flanked by FRT (FLP recognition target) sites. The DNA fragments for introducing the ∆*ssrA*, ∆*intA*‐*ypjF*, and ∆*ssrA*‐*ypjF* mutations were amplified using the primer pairs ON001 and ON002, ON027 and ON028, and ON001 and ON028, respectively, with pCGT1 as the template. Each purified DNA was electroporated into *E. coli* strain BW25113 or MG1655 harboring pKD46, and the transformants resistant to 20 μg/ml chloramphenicol were stored as the ∆*ssrA*, ∆*intA*‐*ypjF*, and ∆*ssrA*‐*ypjF* mutants, respectively.

BW25113 or MG1655 carrying pCY2794 (derivative of pKD46 with *alpA* (Kirby et al., 1994) under the control of the P_BAD_ promoter) was grown in LB medium containing 0.2% arabinose and 100 μg/ml ampicillin at 30°C for 2 h to induce the excision of CP4‐57 from the *E. coli* chromosome. A 2 μl portion of the culture was spread onto an LB agar plate and incubated at 37°C overnight to remove pCY2794. Isolated colonies were streaked onto LB agar plates with or without 100 μg/ml ampicillin to monitor the loss of pCY2794. Complete removal of CP4‐57 was confirmed by PCR, using ON003 and ON225. Plasmids were constructed using standard cloning procedures and Gibson assembly (Gibson, 2011). Detailed schemes for plasmid construction are summarized in Table S2.

### 4.2 Bacterial growth and motility assay

*E. coli* cells were grown in LB medium or MOPS minimal medium (Neidhardt et al., 1974) at 37°C unless otherwise noted. LB containing 1.5% (w/v) agar was used to isolate *E. coli* colonies. Appropriate antibiotics were added to the media. Bacterial growth in a liquid medium was monitored by measuring the OD_660_.

Cell motility was examined with semisolid agar plates (0.5% peptone, 0.3% yeast extract, 0.3% agar). Colonies were inoculated onto the plates and incubated at 30°C for 20 h.

### 4.3 Western blotting analysis

Cell cultures were withdrawn and mixed with an equal volume of 10% of TCA. After standing on ice for at least 10 min, the samples were centrifuged at 15,000 rpm for 3 min at 4°C, and the supernatant was removed by aspiration. Precipitates were washed with acetone by vigorous mixing, centrifuged again, and dissolved in 1× SDS sample buffer (62.5 mM Tris–HCl, pH 6.8, 5% 2‐mercaptoethanol, 2% SDS, 5% sucrose, and 0.005% bromophenol blue) by vortexing for 15 min at 37°C. Prepared cellular extracts were separated by 13 or 15% SDS‐PAGE and subsequently transferred to an Immobilon‐P^SQ^ Membrane (Millipore). The membrane was blocked by 1% (w/v) skim milk in TBS‐Tween (20 mM Tris–HCl, pH 7.5, 150 mM NaCl, 0.1% Tween‐20) at room temperature for 1 h. The membrane was then incubated with TBS‐Tween containing 1% (w/v) skim milk and an antibody (1/10,000 dilution) at room temperature for 1 h. Plasmid‐borne ArfA, ZntR, sfGFP, GST, and endogenous FtsZ were detected by an Anti 6 × Histidine Monoclonal Antibody (9C11, Wako), Monoclonal Anti‐HA clone HA‐7 (Sigma‐Aldrich), Anti‐Green Fluorescent Protein Monoclonal Antibody (mFx75, Wako), GST･Tag Monoclonal Antibody (Sigma‐Aldrich) and Anti‐FtsZ antibody (a gift from Dr. Shinya Sugimoto at Jikei Medical University), respectively. HRP‐conjugated anti‐mouse IgG (Sigma‐Aldrich) and HRP‐conjugated anti‐rabbit IgG (Sigma‐Aldrich) were used as secondary antibodies. Images were visualized and analyzed by a LAS4000 LuminoImager (Fujifilm).

### 4.4 Northern blotting analysis

*E. coli* cells were grown in a LB medium until the OD_660_ reached ~0.5. The expression of *zntR* mRNA or *zntR*‐*arfA* mRNA was then induced by 1 mM IPTG for 15 min. At this point, 200 μg/ml of rifampicin was added to the culture. The culture was harvested at 0, 1, 2, and 4 min after the addition of rifampicin. Total RNA was prepared using the Tripure Isolation Reagent (Roche), according to the supplier's instructions. RNA samples were separated by 1.5% denaturing agarose electrophoresis, transferred onto BrightStar‐Plus Positively Charged Nylon Membranes (Invitrogen), and hybridized with biotinylated oligonucleotides (Integrated DNA Technologies) complementary to the *zntR* or *arfA* mRNA, as shown in Table S4. Hybridization experiments were performed using a NorthernMax kit (Ambion) and a Chemiluminescent Nucleic Acid Detection Module (ThermoFisher Scientific) according to the suppliers' instructions. Images were visualized and analyzed by a LAS4000 LuminoImager (Fujifilm).

### 4.5 Sample preparation for proteomic analysis

Cells were grown in LB medium until the OD_660_ reached ~0.5. After the cells were harvested, they were washed and resuspended with PBS buffer (137 mM NaCl, 8.1 mM Na_2_HPO_4_, 2.68 mM KCl, 1.47 mM KH_2_PO_4_, pH 7.4). The suspension was mixed with an equal volume of 10% of TCA. After standing on ice for at least 10 min, the samples were centrifuged at 15,000 rpm for 5 min at 4°C, and the supernatant was removed by aspiration. Precipitates were washed twice with acetone, by vigorous mixing. Sample preparation for LC–MS/MS was basically performed according to the previous study (Masuda et al., 2009) with some modifications. Proteins were dissolved in PTS solution (12 mM sodium deoxycholate, 12 mM sodium lauryl sulfate, 100 mM Tris–HCl, pH 9.0) and the concentration of the lysate was determined using a Pierce BCA Protein Assay Kit (ThermoFisher Scientific). Subsequently, 50 μg of total protein at a concentration of 1 μg/μl was processed according to the following procedure. First, the lysate was reduced by a treatment with 20 mM dithiothreitol (DTT) at room temperature for 30 min and then alkylated with 50 mM iodoacetamide in the dark at room temperature for 20 min. The protein mixture was then diluted 5‐fold with 50 mM ammonium bicarbonate. For the limited digestion of denatured proteins into peptide fragments, 0.5 μg of Trypsin/Lys‐C Mix (Promega, U.S.A.) was added, and the mixture was incubated at room temperature for 3 h. Subsequently, 1.0 μg of Trypsin/Lys‐C Mix was again added and incubated at 37°C overnight. After the digestion, an equal volume of ethyl acetate and 0.5% trifluoroacetic acid (final concentration) was added. The mixture was shaken vigorously for 2 min and then centrifuged at 15,700 × g for 2 min. The upper ethyl acetate layer was discarded, and the solvent was removed by a centrifugal evaporator. The residual pellet was redissolved in 600 μl of 0.1% TFA and 2% acetonitrile and desalted as follows. The solution was applied to a StageTip composed of an SDB‐XC Empore disk (3 M, U.S.A.), equilibrated with 0.1% TFA and 2% acetonitrile, washed with 0.1% TFA and 2% acetonitrile, and eluted with 0.1% TFA and 80% acetonitrile. The solvent was removed by a centrifugal evaporator, and the residual peptides were redissolved in 120 μl of 0.1% TFA and 2% acetonitrile. This solution was centrifuged at 20,000 × g for 5 min, and a 100 μl portion of the supernatant was collected and subjected to the LC–MS/MS measurement.

### 4.6 LC–MS/MS‐based proteomic analysis

The LC–MS/MS measurements (Gillet et al., 2012) (SWATH‐MS acquisition) were conducted with an Eksigent NanoLC Ultra and TripleTOF 4600 tandem‐mass spectrometer or an Eksigent nanoLC 415 and TripleTOF 6600 mass spectrometer (AB Sciex, U.S.A.). The trap column used for nanoLC was a 5.0 mm × 0.3 mm ODS column (L‐column2, CERI, Japan) and the separation column was a 12.5 cm × 75 μm capillary column packed with 3 μm C18‐silica particles (Nikkyo Technos, Japan). The detailed settings for the LC–MS/MS measurements are shown in Table S5. The SWATH acquisition was performed three times for each sample. Data analysis of the SWATH acquisition was performed using the DIA‐NN software with default settings (Demichev et al., 2020). The library for SWATH acquisition was obtained from the SWATH atlas {http://www.swathatlas.org, the original data are in Midha et al. (2020)}. Only the proteins detected in all three measurements for both samples were used for the fold change calculation. The obtained protein intensities were averaged by using an in‐house R script. The *p*‐value was determined by Welch's t‐test and corrected by the Benjamini‐Hochberg method for multiple comparisons, using the “p.adjust” function in R (for Windows, version 4.1.2).

### 4.7 Sequence analysis

The genetic structure neighboring to *arfA* ORF was done using webFlaGs software (Saha et al., 2020). The program was run in “single sequence” mode to reference the representative database with default parameter settings.

## AUTHOR CONTRIBUTIONS

**Haruka Onodera:** Conceptualization; data curation; formal analysis; investigation; resources; validation; visualization; writing – original draft; writing – review and editing. **Tatsuya Niwa:** Data curation; investigation; methodology; software; supervision; validation; writing – review and editing. **Hideki Taguchi:** Funding acquisition; project administration; supervision; validation; writing – original draft; writing – review and editing. **Yuhei Chadani:** Conceptualization; data curation; formal analysis; funding acquisition; investigation; methodology; project administration; resources; software; supervision; validation; visualization; writing – original draft; writing – review and editing.

## CONFLICT OF INTEREST

The authors declare no competing interest.

## Supporting information


Figure S1

Table S1

Table S2

Table S3

Table S4

Table S5

Table S6

## Data Availability

The MS datasets obtained in this study are deposited in jPOST repository (https://repository.jpostdb.org/preview/13842478476256961e2989a). Sequence files of the plasmids constructed in this study are deposited in the Mendeley Data repository (https://doi.org/10.17632/mmstxfffvk.1).
