# Supplementary material for: Prophage excision switches the primary ribosome rescue pathway and rescue‐associated gene regulations in Escherichia coli
Source: Mol Microbiol. 2022 Dec 5;119(1):44–58. doi: 10.1111/mmi.15003 (PMC10107115; doi:10.1111/mmi.15003)
Supplement: Supplementary file 1 — Figure S1 [file MMI-119-44-s006.pdf]

Supplementary Information for

Prophage excision switches the primary ribosome rescue pathway and rescue-associated gene regulations in *Escherichia coli*

Haruka Onodera, Tatsuya Niwa, Hideki Taguchi and Yuhei Chadani

Yuhei Chadani and Hideki Taguchi
Email: chadani.y.aa@m.titech.ac.jp, taguchi@bio.titech.ac.jp

Supplementary Figures (included in this file)
- Figure S1
- Figure S2
- Figure S3
- Figure S4
- Figure S5

Supplementary Tables (separated in the individual files)
- Table S1
- Table S2
- Table S3
- Table S4
- Table S5
- Table S6

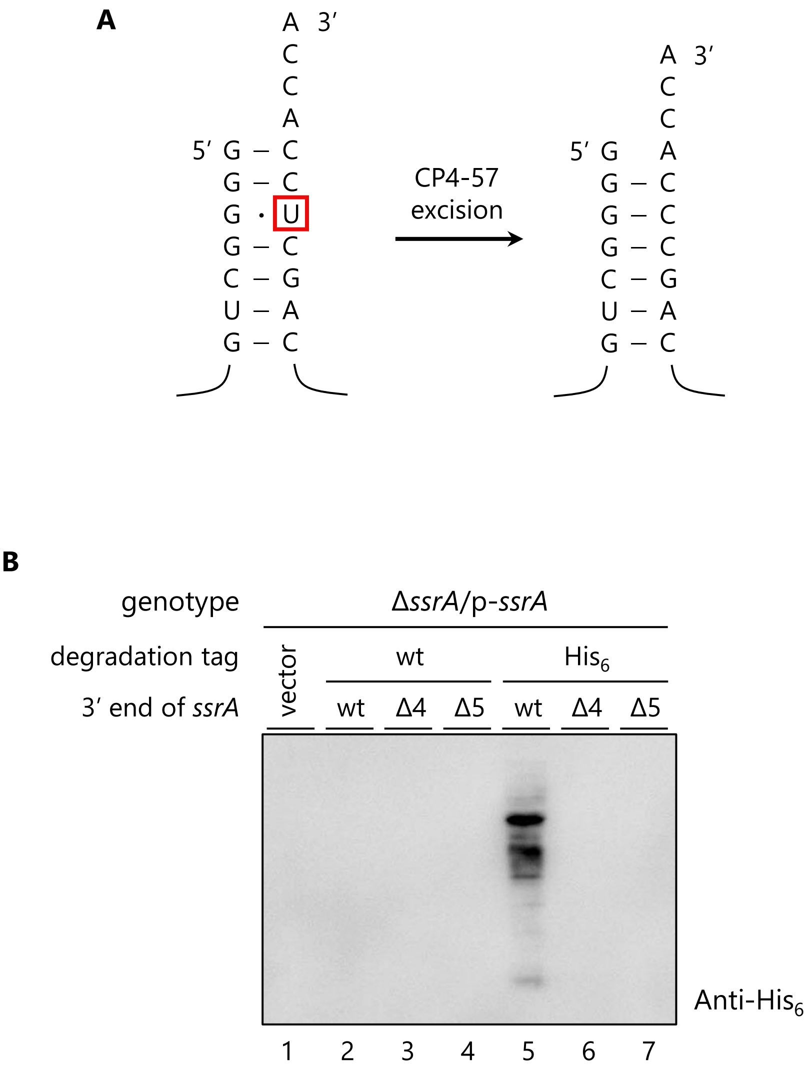

Fig. S1.

(A) The acceptor stem-loop structure at the 3' end of *E. coli* tmRNA and the tmRNAΔU357 mutant. CP4-57 excision deletes the T357 residue of the *ssrA* gene, which forms a G·U wobble base pair in the acceptor stem in the tRNA-like domain.

(B) Inactivation of $tmRNA^{His}$ by prophage excision-introduced mutations in other bacterial species. The *E. coli* Δ*ssrA* strain harboring pMW118 (vector control, lane 1) and its derivatives, p-*ssrA* (lane 2), p-*ssrA*Δ4 (induced by the excision of Ype11X in *Yersinia pestis* CO92, which deletes four nucleotides at the 3' end, lane 3), p-*ssrA*Δ5 (induced by the excision of Stm27X in *Salmonella enterica* serovar Typhimurium LT2, Eco48X in *Escherichia coli* RS218 and Oi108 in *Escherichia coli* O157:H7 EDL933, which deletes five nucleotides at the 3' end, lane 4), p-$ssrA^{His}$ (lane 5), p-$ssrA^{His}$Δ4 (lane 6) and p-$ssrA^{His}$Δ5 (lane 7) were grown in LB medium until mid-log phase. Cellular extracts were fractionated by SDS-PAGE and analyzed by western blotting using an anti-His_6 antibody.

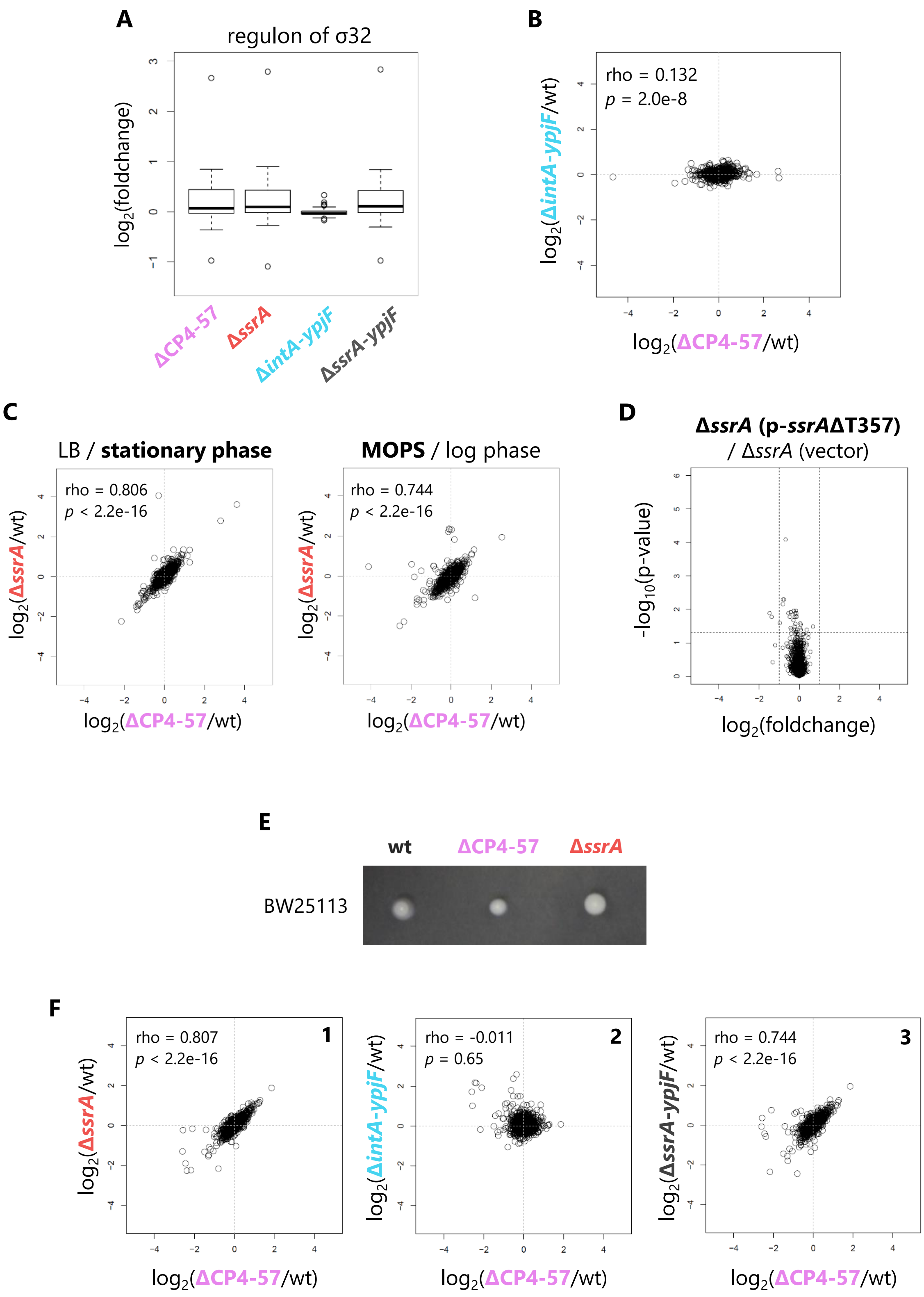
A
regulon of σ32
log2(foldchange)
ΔCP4-57
ΔssrA
ΔintA-ypjF
ΔssrA-ypjF
B
rho = 0.132
p = 2.0e-8
log2(ΔintA-ypjF/wt)
log2(ΔCP4-57/wt)
C
LB / stationary phase
rho = 0.806
p < 2.2e-16
log2(ΔssrA/wt)
log2(ΔCP4-57/wt)
MOPS / log phase
rho = 0.744
p < 2.2e-16
log2(ΔssrA/wt)
log2(ΔCP4-57/wt)
D
ΔssrA (p-ssrAΔT357)
/ ΔssrA (vector)
-log10(p-value)
log2(foldchange)
E
wt
ΔCP4-57
ΔssrA
BW25113
F
1
rho = 0.807
p < 2.2e-16
log2(ΔssrA/wt)
log2(ΔCP4-57/wt)
2
rho = -0.011
p = 0.65
log2(ΔintA-ypjF/wt)
log2(ΔCP4-57/wt)
3
rho = 0.744
p < 2.2e-16
log2(ΔssrA-ypjF/wt)
log2(ΔCP4-57/wt)

Fig. S2.

(A) Fold change values of the heat shock regulons (σ32) were extracted from the results in **Fig. 2**, and represented as a boxplot.

(B) Two-dimensional plots of the fold change values in the *E. coli* BW25113 ΔCP4-57 (horizontal axis) and Δ*intA*-*ypjF* strains (vertical axis). The plot is represented with Spearman's rho and *P*-values calculated by Spearman's rank correlation tests.

(C) Two-dimensional plots comparing the proteomic rearrangements in the BW25113 ΔCP4-57 and BW25113 Δ*ssrA* strains in LB medium at the stationary growth phase (left) or in MOPS minimal medium at the mid-log growth phase (right). Cells were grown at 37 °C in the indicated medium and then subjected to the SWATH-MS analysis, as shown in **Fig. 2**. Each plot is represented with Spearman's rho and *P*-values calculated by Spearman's rank correlation tests.

(D) A volcano plot showing the poor proteomic change in the Δ*ssrA* strain expressing tmRNAΔU357, compared to the Δ*ssrA* strain harboring an empty vector. Fold change and *P*-values of each protein are represented by dots and plotted according to its $\log_2$ fold change on the horizontal axis and *P*-value on the vertical axis. The lines indicate a *P*-value of 0.05 and 2-fold change.

(E) Swimming motility of BW25113 wild-type strain and its derivatives, ΔCP4-57 and Δ*ssrA* mutant. Colonies were inoculated onto semisolid agar plates and incubated at 30 °C for 20 hrs.

(F) Two-dimensional plots of the fold change values in the *E. coli* MG1655 mutant indicated below and on the side of the graph. Each plot is represented with Spearman's rho and *P*-values calculated by Spearman's rank correlation tests.

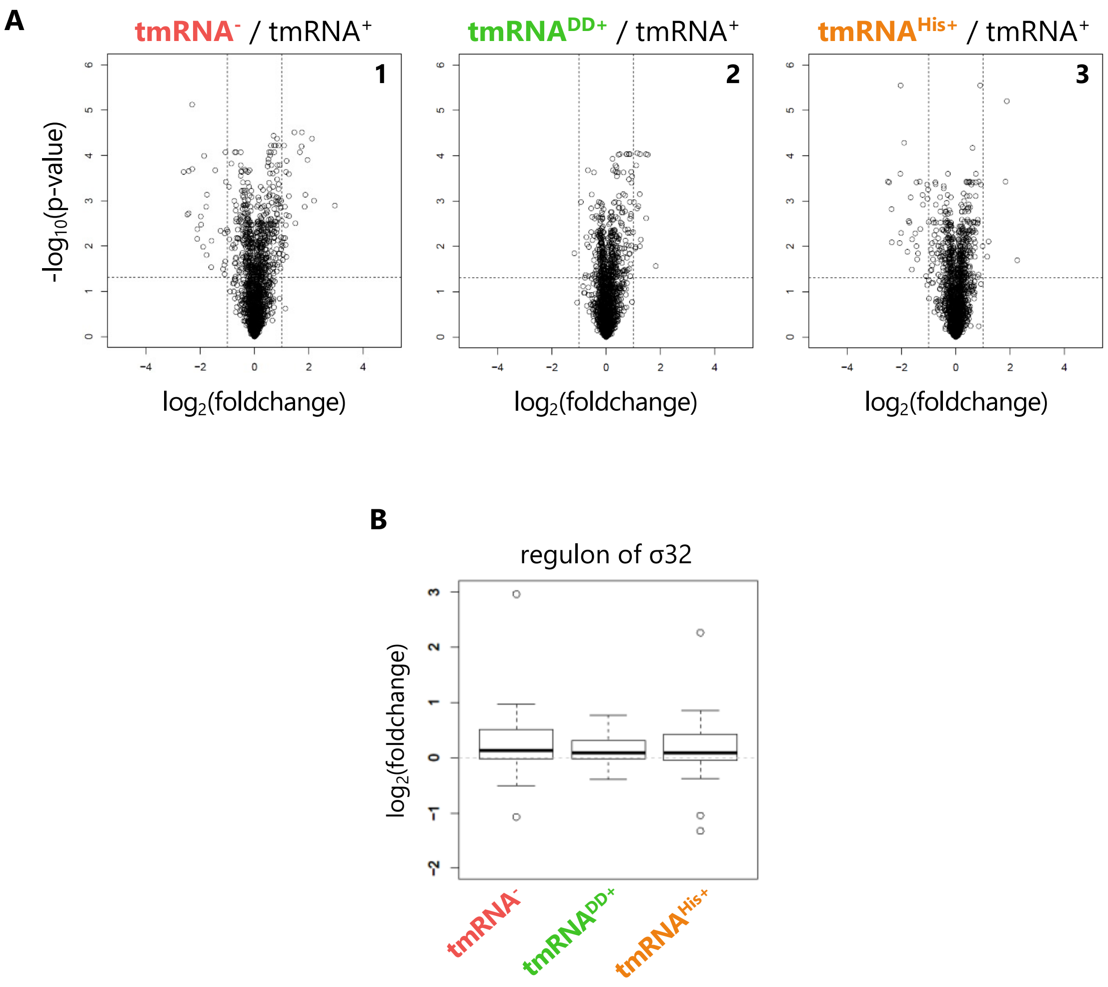

Fig. S3.

(A) Proteomic rearrangements in proteolysis-deficient tmRNA-expressing strains. The fold change relative to the wild-type tmRNA expressing strain and the *P*-value of each protein in the Δ*ssrA* strain harboring pMW118 (panel 1) and its derivatives, p-$ssrA^{DD}$ (panel 2) and p-$ssrA^{His}$ (panel 3), are represented by volcano plots, as shown in **Fig. 2**. (B) Fold change values of the heat shock regulons (σ32) were extracted from the results in **Fig. 3**, and are represented by boxplots.

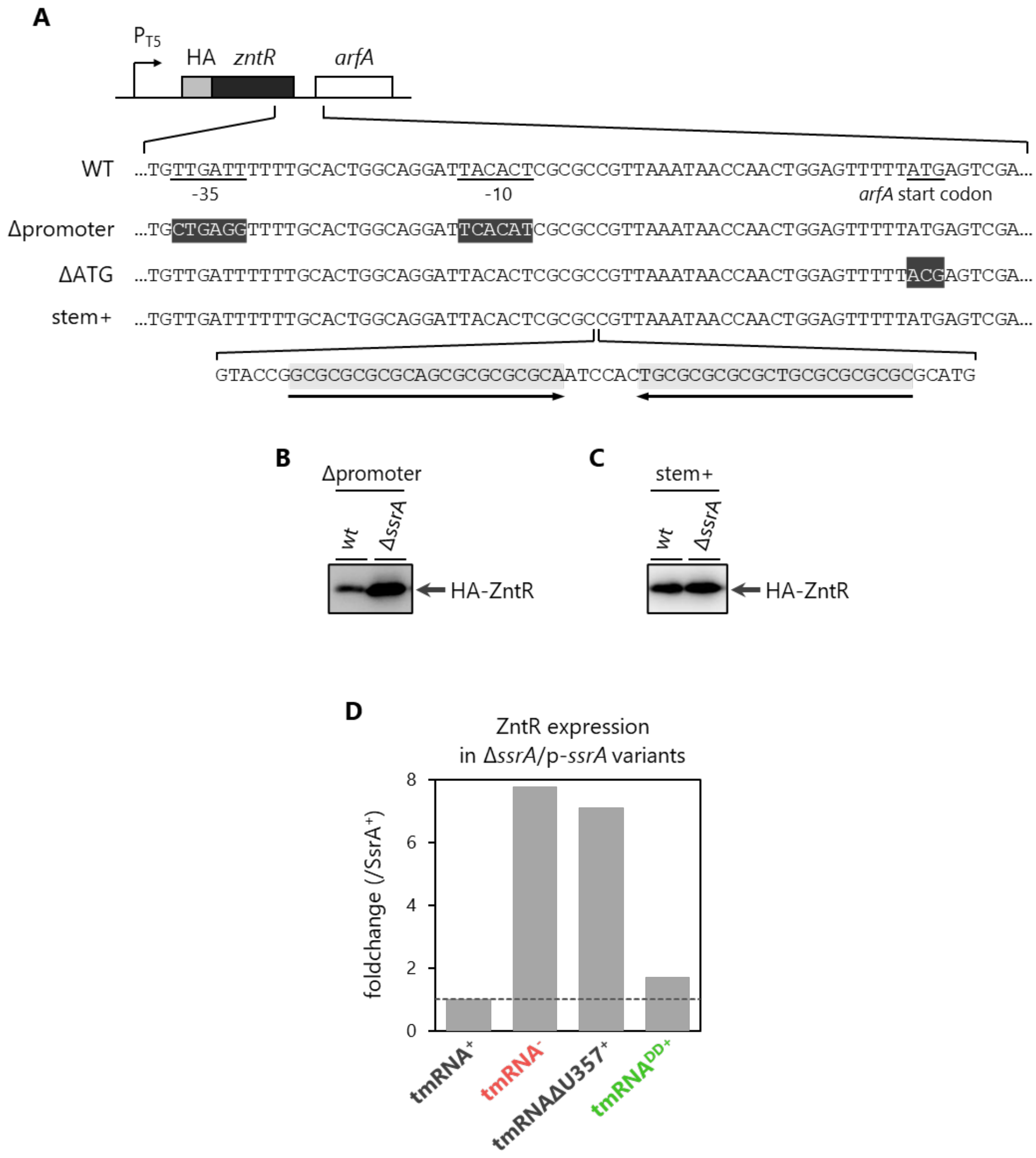

Fig. S4.

(A) Schematic representation of the mutations introduced into the *zntR-arfA* operon: WT (wild-type), ΔATG (disruption of the initiation codon of *arfA*), Δpromoter (disruption of *arfA* promoter) and stem+ (insertion of an artificial stem-loop between *zntR* and *arfA,* indicated by arrows).

(B) Expression of HA-tagged ZntR from *zntR-arfA* lacking the *arfA* promoter. BW25113 and the Δ*ssrA* strain harboring pOH020 (*zntR-arfA* Δpromoter) were grown in LB until the OD_{660} reached 0.2-0.3. IPTG (100 μM) was then added to induce the expression of

zntR, and cells were grown until the OD_{660} doubled. Cellular extracts were prepared and analyzed as in **Fig. 4D**.

(C) Expression of HA-tagged ZntR from *zntR-arfA* with the insertion of an artificial stem-loop. BW25113 and the Δ*ssrA* strain harboring pOH021 (*zntR-arfA* stem+) were grown and analyzed as in **Fig. 4D**.

(D) Expression levels of ZntR in tmRNA variant-expressing cells, extracted from the SWATH-MS analyses in **Fig. 3**. The dashed line indicates a fold change value of 1.

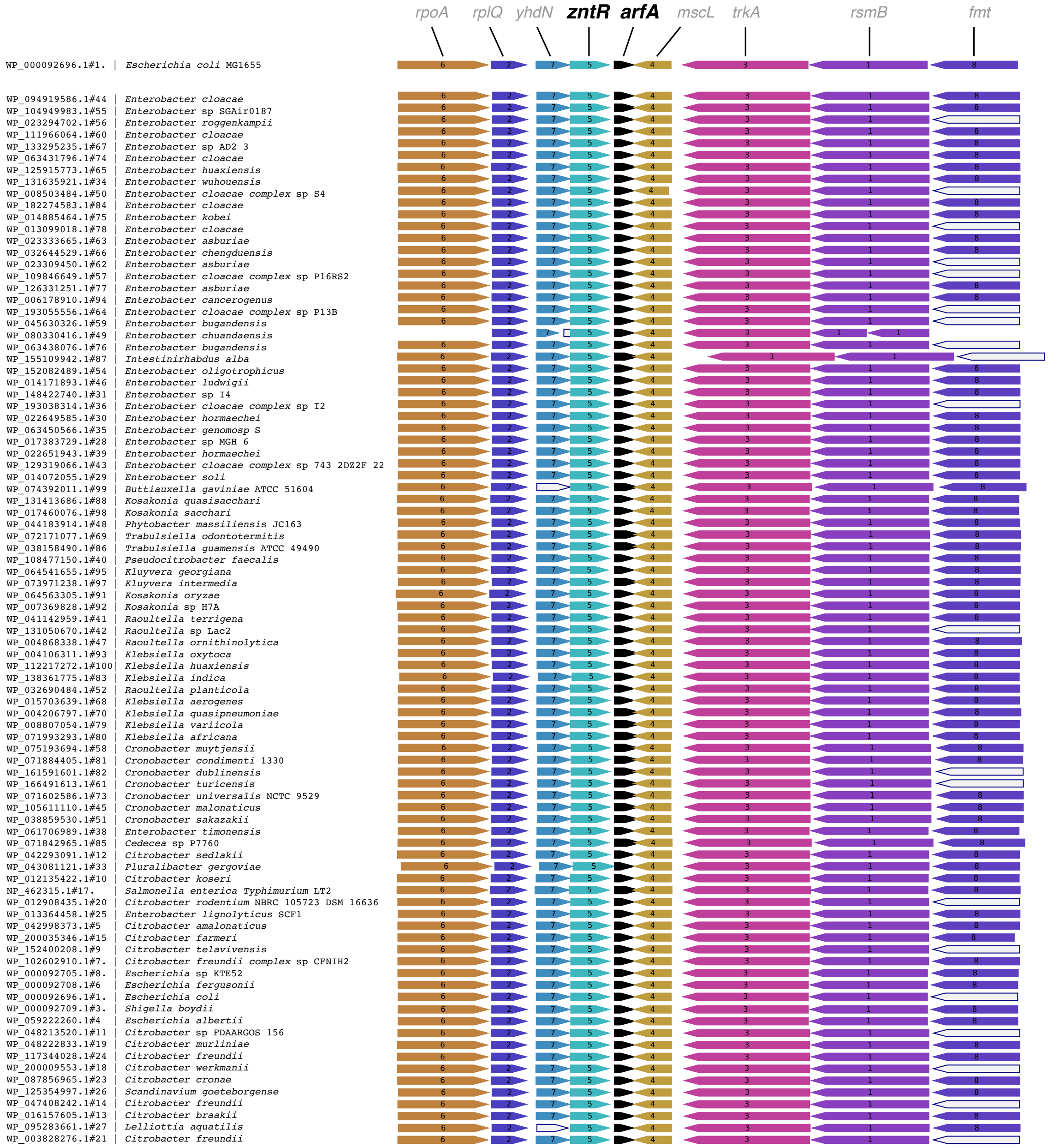

Fig. S5.

The genetic structure of *arfA*-surrounding genes among enterobacteria described by webFlaGs software (Saha *et al*., 2020). Arrow boxes from 1 to 8 indicate the ORFs homologous to *rpoA* (no.6), *rplQ* (no. 2), *ydhN* (no.7), *zntR* (no.5), *mscL* (no. 4), *trkA* (no. 3), *rsmB* (no. 1) and *fmt* (no.8), respectively. Colorless arrowed boxes without the number indicates the ORFs that do not exist in *E. coli* MG1655 strain.
